# Advances and Applications of Brain Organoids

**DOI:** 10.1007/s12264-023-01065-2

**Published:** 2023-05-24

**Authors:** Yang Li, Peng-Ming Zeng, Jian Wu, Zhen-Ge Luo

**Affiliations:** School of Life Science and Technology, ShanghaiTech University, Shanghai, 201210 China

**Keywords:** Human pluripotent stem cell, Brain organoid, Brain development, Neurological disease

## Abstract

Understanding the fundamental processes of human brain development and diseases is of great importance for our health. However, existing research models such as non-human primate and mouse models remain limited due to their developmental discrepancies compared with humans. Over the past years, an emerging model, the “brain organoid” integrated from human pluripotent stem cells, has been developed to mimic developmental processes of the human brain and disease-associated phenotypes to some extent, making it possible to better understand the complex structures and functions of the human brain. In this review, we summarize recent advances in brain organoid technologies and their applications in brain development and diseases, including neurodevelopmental, neurodegenerative, psychiatric diseases, and brain tumors. Finally, we also discuss current limitations and the potential of brain organoids.

## Introduction

The development of the nervous system is a strictly orchestrated spatial and temporal process that generates an immense diversity of cell types. Understanding the basic mechanisms of human brain formation and disorder is important because of the widespread health burden of neurological disorders. The human brain differs significantly from model animals like mice in terms of size, surface area, and the complexity of cytoarchitecture [1, 2]. Due to the limited accessibility of live human brains and inconsistent treatment of post-mortem or surgically removed human brain samples, it is difficult to replicate data and apply it to clinical treatment.

Human pluripotent stem cells (hPSCs), including human embryonic stem cells (hESCs) and human induced pluripotent stem cells (hiPSCs), which possess a unique ability for self-renewal and broad plasticity for differentiation, have emerged as invaluable tools for exploring the human brain. By providing different levels of inhibition of bone morphogenic protein (BMP) and transforming growth factor-β/NODAL signaling, known as “Dual SMAD” inhibition, hPSCs can be induced into neural stem cells and cortical pyramidal neurons, among others [3]. This method obtains highly consistent neural cells *in vitro*. However, monolayer cultures lack cell-type diversity and spatial complexity, and cannot recapitulate cell-cell interactions and certain important cellular properties, such as cell polarity and guided cell migration. In 2008, *in vitro* neural differentiation was further improved using more reproducible serum-free methods, known as SFEBq (serum-free, floating culture of embryoid body (EB)-like aggregates) [4]. Aggregates plated on coated dishes would efficiently differentiate into multiple small rosettes of neural precursors surrounding and growing around apical lumens. Afterward, aggregates embedded in Matrigel formed an organized architecture, referred to as a brain organoid, mimicking the cellular and structural complexity of the prenatal developing brain and the radial migration of later-born neurons from the ventricular zone in the center of the brain out to the superficial layers [5]. The advent of brain organoid technology has brought a new era of human brain research. Brain organoids have been used to model human-specific developmental processes and recapitulate disease-specific pathologies associated with neurodevelopmental, neuropsychiatric, and neurodegenerative disorders [2, 6–8]. In this review, we discuss the development of brain organoid culture systems and their applications to various neurological disorders, including neurodevelopmental disorders, neurodegenerative diseases, major psychiatric diseases, and brain tumors.

## Development of the Brain Organoid System

Human brain development begins around the third week of gestation, when the neural tube forms, and differentiation occurs along the anterior-posterior axis. By the sixth gestational week, the difference in the rates of proliferation of cells in rostral regions of the neural tube results in the formation of three brain vesicles, the prospective forebrain, midbrain, and hindbrain [9]. These basic structures provide the basis for the progressive development of regionally more defined brain regions such as the cerebral cortex and thalamus (forebrain), parts of the brainstem (midbrain), and the pons and cerebellum (hindbrain) [9]. To reconstruct the developmental processes of the human brain *in vitro*, researchers have established various culture systems for the whole brain and specialized regions of the mature central nervous system, such as the forebrain organoid, midbrain organoid, and hindbrain/spinal cord organoid [10].

**Fig. 1:**
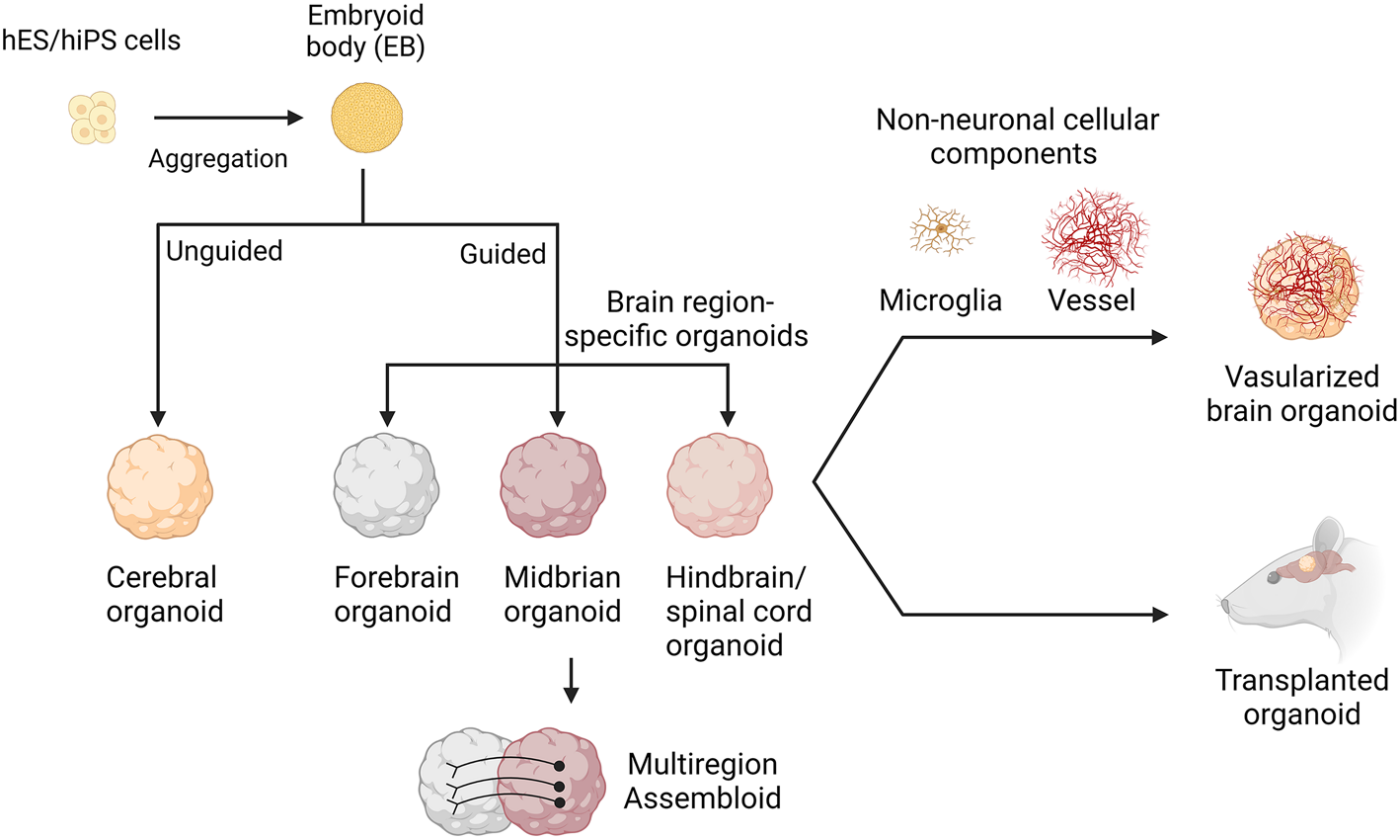
Advances in brain organoid methods. Human stem cell/pluripotent stem cells can be differentiated in self-organizing 3D cultures to derive unguided neural organoids (cerebral organoids) or brain region-specific organoids resembling various regions of the nervous system. Brain region-specific organoids can be combined to generate assemblies to model complex cell-cell interactions and neural circuit formation in the human nervous system. Brain organoids also can be fused with non-neuronal cellular components such as vessels and microglia or transplanted into animals to vascularize brain organoids. The figure was created with BioRender.com.

The method of forming the whole brain is referred to as the unguided method (Fig. 1). This protocol was developed by Lancaster *et al* [5], who embedded EBs in Matrigel and cultured them in a neural induction medium without the use of patterning growth factors, focusing instead on improving growth conditions and providing the environment necessary for intrinsic cues to help self-organization. Taking advantage of this method, embryonic stem cells or human induced pluripotent stem cells (hiPSCs) can self-assemble to form cerebral organoids, including various discrete, but interdependent brain regions (Fig. 1). This can be used to study the interactions of multi-regional brain areas, but is less subject to external control and more stochastic [5, 11]. Other techniques for developing specialized brain regions are known as guided approaches, which involve the addition of small molecules to organoids to steer them in a certain direction [11]. To promote specific neural fates that generate organoids with varied identities from the forebrain to the midbrain to the hindbrain, defined developmental patterning cues are used, among which some have previously been effectively used in 2D differentiation procedures [3]. Dorsal forebrain organoids can be developed after continuous “Dual SMAD” inhibitor induction, and a sonic hedgehog antagonist is added after the SMAD inhibitor to induce ventral forebrain [12–15]. Functional hippocampal granule- and pyramidal-like neurons have been generated *via* long-term dissociation culture of the self-organizing dorsomedial telencephalic tissues derived from hESCs treated with Wnt agonist and BMP ligand, under optimized culture and treatment conditions [16]. After receiving initial dual SMAD inhibitors, EBs have been preprogrammed to have a neuroectodermal fate. Insulin and MAPK/ERK inhibitors were then used to prevent over-causation to a midbrain fate, and the addition of BMP7 guided toward thalamus tissue development [17]. The forebrain organoids are similar to the human cerebral cortex in the aspects of cell types and structures, containing several ventricular structures, each with a defined ventricular zone enriched for FOXG1^+^ forebrain progenitors and also producing astrocytes at a later stage of development [13]. Many cortical organoid techniques have poor lamination and layer distinction, but some have demonstrated the sequential production of neurons expressing layer-specific markers in the right order, including RELN, TBR1, CTIP2, and SATB2 [5]. The ability of brain organoids to mimic fetal brain development in utero has been tested by transcriptome sequencing, single-cell sequencing, and epitranscriptome analysis of multi-period organoids [18, 19]. Several studies have examined the physiological characteristics of neurons produced using organoid methods and demonstrated how their functional development proceeds through time [5, 20, 21]. Electrophysiological recordings and Ca^2+^ surges have shown that neurons produced in brain organoids functionally mature gradually and fire spontaneously [5, 13]. The frequency of this firing is sensitive to the application of glutamate and glutamate receptor antagonists, indicating the presence of glutamatergic neurons [13].

Although spinning bioreactors have been widely used to help increase nutrient and oxygen diffusion within organoids by agitating and better circulating the medium, the small contact surface of the organoid with the culture medium has the disadvantage that the inside cells die at a later stage of culture. Different techniques such as sectioning, adding vessel-like structures, and orthotopic xenotransplantation, have been used to extend the culture time of the organoid [21–25] (Fig. 1). Organoids cultured at the air-liquid interface were able to show a great improvement in the survival and maturation of neurons [21, 22]. Some studies co-cultured hiPSC-derived endothelial cells or umbilical vein endothelial cells with brain organoids and reported robust engraftment with the formation of capillary-like structures [23–25]. Recently, we generated a human brain vessel organoid, which possesses multiple cell types including pericytes, endothelial cells, and microglia, and has a vascular lumen structure [25]. By encapsulating them with brain organoids, the fused organoids not only provided a variety of cell types but also reduced the apoptosis, and increased the neural progenitor proliferation and cortical thickness [25]. An alternate strategy engineered hESCs to ectopically express human ETV2 to create endothelial cells within cortical organoids, resulting in the appearance of vascular network-like structures along with enhanced neuron maturity [26]. In addition, some studies have transplanted organoids into rodent brains so that the implanted organoids can integrate into the host brain circuits to extend the cultural life of organoids with the production of more mature functional neurons [27–31] (Fig. 1).

Organoids that are patterned to particular brain regions contain more homogeneous populations of progenitor cells and neurons, which minimizes inter-organoid variation. However, this enhancement also reduces the possibility of investigating the interactions between various brain regions. To overcome this limitation, several studies have created assembled organoids, named assemblies. which are formed by fusing organoids from different regions. Ventral forebrain-like organoids containing gamma-aminobutyric acid (GABA)-ergic cortical interneurons can be combined with dorsal forebrain-like organoids in order to capture the cell-cell interactions *in vitro* [32, 33] (Fig. 1). The construction of a thalamus-cortex assembly has modeled the connection loops between the cortex and the thalamus [17]. Recently, Jimena *et al.* have created a cortico-spinal-muscle assembly to model multi-organ connections with functional regulatory effects [34]. In this system, glutamate uncaging or optogenetic stimulation of cortical spheroids can trigger strong muscle contraction, and these assemblies are morphologically and functionally intact for up to 10 weeks post-fusion [34].

The nervous system contains not only cells of neuroectodermal origin but also microglia from the yolk sac and vascular cells from the mesoderm. Microglia are the brain’s resident immune cells that play crucial roles in regulating neuronal circuits, preserving homeostasis, and monitoring the surrounding area [35]. Due to the diverse germ layer origins, it has been challenging to generate brain organoids containing blood vessels and microglia. Some studies have tried to add *in vitro* cultured microglia-like cells to brain organoids [36–38]. Recently, we developed a new strategy for vascularizing brain organoids [25]. First, brain vessel organoids were generated by sequential mesodermal and endothelial cell induction and then fused with brain organoids at an early stage. The fused organoids formed brain-blood barrier (BBB)-like structures and contained extensive amounts of microglia, which responded to immune stimuli and engulfed synapses [25]. The current induction of blood vessels and microglia *in vitro* does not fully mimic the *in vivo* situation, and more work is needed to further optimize the induction conditions of the organoid to make its development more stable and mature.

## Neurodevelopmental Disorders

Most neurodevelopmental disorders occur with complex conditions in childhood and remain incurable and irreversible [39, 40]. Limited research models and poor diagnostic conditions and standards render neurodevelopmental disorder studies at a bottleneck stage. The advent of brain organoid models offers an opportunity to uncover unknowns and develop new intervention strategies [41].

**Fig. 2:**
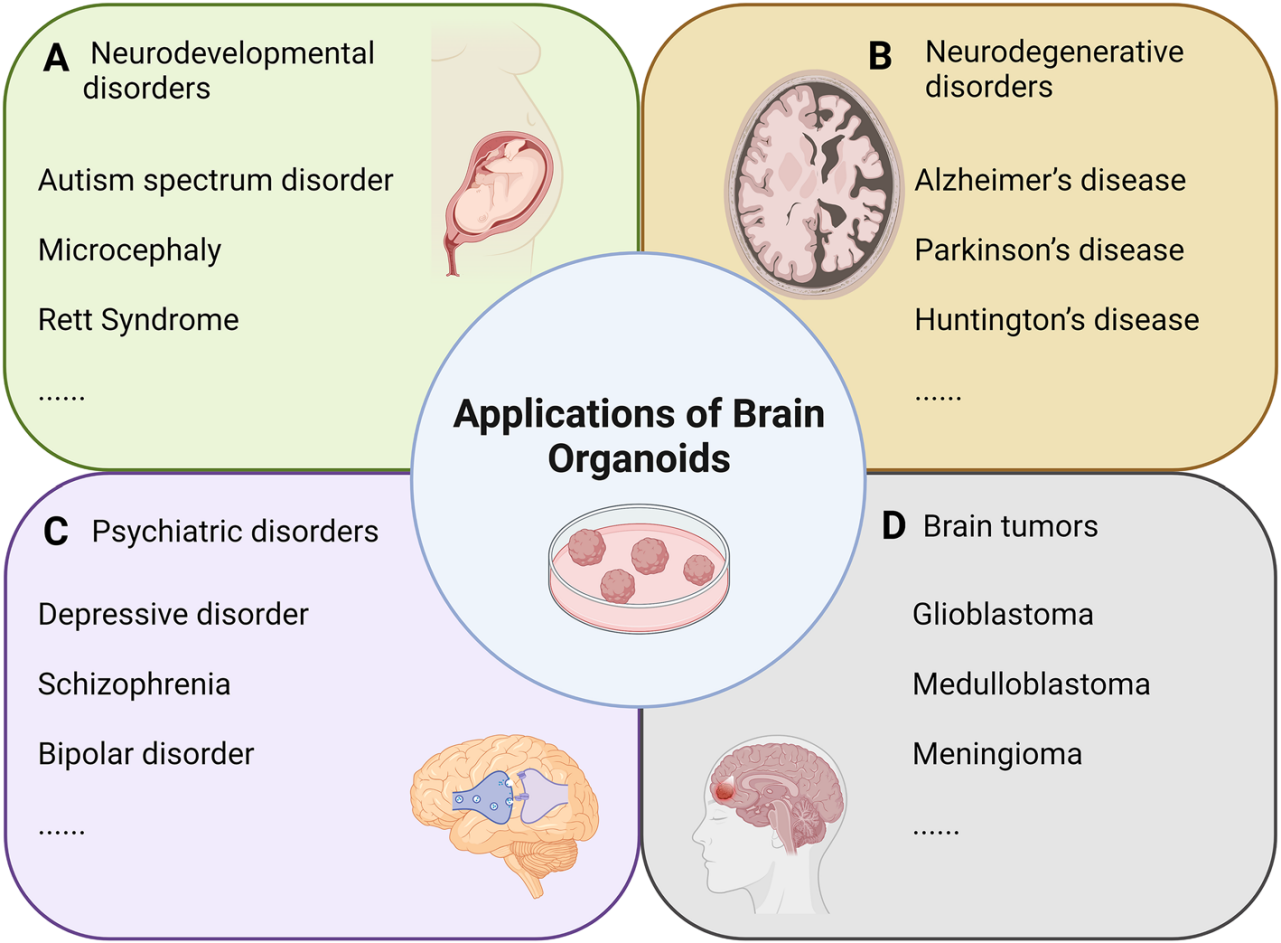
Applications of brain organoids. **A** Neurodevelopmental disorders. Brain organoids have been used to study neurodevelopmental disorders such as autism spectrum disorder, microcephaly, and Rett syndrome. **B** Neurodegenerative disorders. iPSC-induced or CRISPR-Cas9 gene-edited brain organoids have been successfully established for studying aging-dependent Alzheimer’s disease (AD), Parkinson’s disease (PD), and Huntington’s disease (HD). **C** Psychiatric disorders. Mental diseases mainly include depressive disorder, schizophrenia, and bipolar disorder. **D** Brain tumors. They also provide a unique opportunity to model brain tumors such as glioblastoma, medulloblastoma, and meningioma. The figure was created with BioRender.com.

Autism spectrum disorder (ASD) is considered to result from overall brain developmental defects, especially at the synapse level, and genetic and environmental factors can both lead to the pathologies of ASD [42] (Fig. 2). As a genetically heterogeneous syndrome, ASD is found in patients with fragile X syndrome, tuberous sclerosis, Joubert syndrome, and Rett syndrome, among others. Besides, genomic copy-number variants are also closely associated with ASD such as deletions and duplications of chromosome 16p11.2 and duplications of maternal 15q11-q13 [43].

The induced neural cells from hiPSCs of ASD patients in a 2D culture system have been used to study the molecular and cellular mechanisms of ASD. For example, hiPSC-derived neuronal progenitor cells (NPCs) from ASD patients with megalencephaly have been found to have a greater ability of proliferation regulated by β-catenin/BRN2 transcriptional activity [44]. Furthermore, ASD-derived neurons display decreased synaptogenesis and defects in neuronal networks [44]. Co-culture of ASD-derived astrocytes and neurons has shown that astrocytes play a negative role in neuronal morphology and synaptogenesis *via* the production of reactive oxygen species and the cytokine interleukin-6 [45]. These 2D culture models provide some clues about the relationships between neuronal cell types in ASD. Nevertheless, as a heterogeneous disorder, limited cell types and a lack of brain environment restrict the usage of 2D culture systems. The emergence of organoid models brings a new opportunity for deeper mechanistic understanding.

The hiPSC-derived telencephalic organoid model has been developed to study severe idiopathic ASD [46]. Using this model, it has been revealed that the transcription factor FOXG1 dysregulates the proliferation and differentiation of GABAergic inhibitory neurons, and the imbalance of GABA/glutamate neuronal fates can be reversed by the knockdown intervention of FOXG1 [47]. The single-nucleotide mutation of the *CDH8* gene has been associated with ASD [43]. The cerebral organoids derived from CDH8^+/−^ iPSCs exhibit large-scale overlapped differentially-expressed genes (DEGs) with the transcriptome of idiopathic ASD organoids [48]. Non-coding RNA *DLX6-AS1* is dramatically upregulated in these ASD organoids and essential for GABAergic interneuron differentiation [48]. ASD forebrain organoids have also revealed the heterochronicity of developmental gene networks, which are associated with morphological growth acceleration [49]. The cortical organoid with haploinsufficiency of three ASD risk genes *SUV420H1*, *ARID1B,* and *CHD8* displays phenotypic convergence with the asynchronous development of GABAergic neurons and deep-layer excitatory projection neurons through distinct molecular pathways, which leads to abnormal circuit activity [50].

Microcephaly is a kind of neurodevelopmental disorder with a smaller brain size in patients [51] (Fig. 2). Several genes have been shown to be linked with microcephaly, such as *MCPH1, WDR62, CDK5RAP2, CEP152,* and *ASPM* [52–54]. Knockdown of the *CDK5RAP2* gene in cerebral organoids causes premature neural differentiation and leads to a marked reduction in organoid size, and over-expression of *CDK5RAP2* can rescue these defects. Mutation of Centrosomal-P4.1-associated protein (CPAP) leads to Seckel syndrome with microcephaly [55]. Cerebral organoids with the natural CPAP microcephaly mutation show smaller size caused by depletion of the cortical neural progenitor radial glial cells (RGCs), and early neuronal differentiation, probably *via* the action of the cilium disassembly complex [55]. Asparaginyl-tRNA synthetase1 (NARS1) has also been reported to be a risk gene associated with microcephaly [56]. Cortical organoids induced by patients with the NARS1 mutation exhibit reduced proliferation of RGCs, impaired differentiation, and smaller sizes, indicating that NARS1 is important for normal human brain development [56]. By using brain organoid models, several studies have investigated the effects of Zika virus (ZIKV) infection, which is associated with the occurrence of microcephaly in newborns, on brain development and neural precursor cell proliferation [20, 57]. Human neural progenitors, neurospheres, and brain organoids infected with ZIKV exhibit reduced growth and increased cell death [58]. The immune receptor Toll-like-receptor 3 and downstream pathways are activated by ZIKV, thus affecting neurogenesis and resulting in apoptosis [59]. Taken together, brain organoid models have faithfully mimicked brain developmental phenotypes and offer a platform for exploring the molecular basis of microcephaly.

Rett Syndrome (RTT) is another type of neurodevelopmental disorder caused by the mutation of X-linked methyl-CpG binding protein 2 (*MECP2*), leading to mental retardation primarily in females [60] (Fig. 2). Before the appearance of brain organoid models, it was difficult to study the dynamic molecular and cellular processes due to limited patient samples and unreliable mouse models. Benefiting from 3D brain organoids, more molecular features of RTT have been revealed [61]. For example, *MECP2-*deficient or mutant cerebral organoids show defects in early neurogenesis and increased expression of miR-199 and miR-214 [61]. Interestingly, the defects in neural development can be rescued by the down-regulation of these miRNAs. Another study showed that *MECP2* mutant human interneurons (INs) are abnormal and identified an epigenetic reader BRD4 as a trigger of IN dysfunction [62]. Thus, brain organoids provide an opportunity for the identification of targets for RTT therapy.

Fragile X syndrome (FXS) is an X-linked dominant disorder caused by the low expression of the *FMR1* gene due to excessive CGG repeats in its 5′ untranslated region [63, 64]. The encoded protein FMRP inhibits the translation of specific mRNAs and thus decreased FMRP protein incurs an excess of translated products, which further affects neuronal maturation and synaptic plasticity [63, 64]. The 2D culture system has been applied to the study of FXS and found that decreased expression of *FMR1* leads to poor neuronal maturation [65, 66]. Brain organoids derived from *FMRP*-KO iPSCs showed bigger sizes and an increased number of glial cells [67]. In addition, FXS forebrain organoids from patient-derived iPSCs exhibit reduced neural progenitor proliferation, dysregulated differentiation, increased synapse formation, hyperexcitability, and a deficit in the production of GABAergic neurons [68]. Interestingly, pharmacological inhibition of the phosphoinositide 3-kinase pathway rescues the neurodevelopmental and synapse formation defects in FXS forebrain organoids [68].

Down syndrome (DS) is caused by the presence of an additional copy of *Homo sapiens* chromosome 21 (HSA21), termed trisomy 21 [69, 70]. The prevalence of DS is around 0.125% worldwide [69]. DS patients usually have intellectual disabilities and other defects of bodily systems such as the musculoskeletal and cardiovascular systems [69, 70]. By adding a partial trisomy of *Mus musculus* chromosome 16 (MMU16) that is orthologous to HSA21 in humans, several DS mouse models have been established [70, 71]. However, these models are artificial and cannot reflect the true DS pathologies because of the discrepancies between humans and mice. Xu *et al.* [72] have established DS human brain organoids and found overproduction of OLIGO2^+^ neural progenitors, which leads to excessive GABAergic interneuron production. Another study focused on microglia in DS organoids and found that tau protein triggers microglial interferon (IFN)-I signaling, which causes increased synaptic pruning, and the microglia dysfunction and senescence can be rescued by inhibiting the IFN-I receptor [73]. Interestingly, DS cerebral organoids show increased DSCAM/PAK1 pathway activity and down-regulation or inhibition of this pathway reverses the abnormal neurogenesis in DS organoids [74].

Angelman syndrome (AS) is a rare neurodevelopmental disorder with 0.4% prevalence, caused by the loss of UBE3A protein, an E3 ubiquitin ligase in neurons [75, 76]. Notably, AS human brain organoids exhibit early silencing of paternal UBE3A and abnormal neuronal activity, which are partially rescued by topoisomerase inhibitors [77]. Using organoid systems, it has been revealed that UBE3A suppresses neuronal excitability *via* ubiquitin-mediated degradation of calcium- and voltage-dependent big potassium (BK) channels, providing mechanistic insights into AS occurrence [78].

Besides, brain organoids have also been used to model other neural developmental disorders, such as macrocephaly [79, 80], neuronal heterotopia [81, 82], tuberous sclerosis [83, 84], and Timothy syndrome [85]. These studies have revealed the molecular basis of these syndromic disorders, leading to the identification of intervention targets or the development of strategies to restore deficits in the context of disease.

## Neurodegenerative Disorders

Neurodegenerative diseases (NDDs) are major threats to human health caused by the progressive death of selectively vulnerable populations of neurons and the loss of normal brain functions [86]. Major types of NDD include Alzheimer’s disease (AD), Parkinson’s disease (PD), amyotrophic lateral sclerosis (ALS), spinal muscular atrophy, Batten disease, multiple sclerosis, and Huntington’s disease (HD) (Fig. 2). Aging, genetics, protein misfolding, and programmed cell death are the main causal factors of NDDs [87–89]. Patients with NDDs have various clinical manifestations including memory loss, cognitive dysfunction, and abnormalities in behavior, language, and respiration, which severely affect the normal life of patients and endanger their safety [90–92]. NDDs are in urgent need of effective treatment programs and drugs, which require a deep understanding of the pathogenesis.

Various animal models have been used to investigate the pathogenesis of NDDs [93–96]. However, due to the large inherent differences between human and animal models, the value of the results based on model systems is suboptimal. Because of the rarity and difficulty in obtaining human tissues, some studies have used stem cell induction technology *in vitro* to induce neurons and glial cells for the mechanistic investigation of NDDs [97, 98]. However, it is difficult to simulate the complex environment inside the real human brain for the induced single cell types, so some studies are beginning to use organoid technology to study related NDDs by establishing organoids representing different brain regions, such as the whole brain, forebrain, midbrain, striatum, and sensorimotor cortex, derived from patient iPSCs [99–104].

AD is the most common neurodegenerative disease [90], with pathological features mainly including amyloid plaques formed by the accumulation of extracellular amyloid-beta (Aβ) and intracellular neurofibrillary tangles formed by the accumulation of phosphorylated tau protein [105]. Brain organoids can be induced from hiPSCs from patients with familial AD (*APP*, *PSEN1*, and *PSEN2* mutations) or patients with sporadic AD, leading to the identification of modulators of the tau interactome, reproduction of AD pathology, and findings of cell fate changes in AD organoids [99–101]. Apolipoprotein E4 (APOE4) is the strongest genetic risk factor associated with late-onset AD among the three polymorphic alleles (*APOE2*, *APOE3*, and *APOE4*), and has recently been proposed to impair myelination *via* cholesterol dysregulation in oligodendrocytes [106, 107]. Interestingly, APOE4 organoids exhibit more severe synaptic loss and neurodegeneration phenotypes [108]. Cerebral organoids increase Aβ and p-Tau by inducing beta-secretase 1 and glycogen synthase kinase-3 alpha/beta levels after being exposed to serum from AD patients [109]. By integrating mathematical modeling and the pathological features of AD in iPSC-derived cerebral organoids, a high-content screening platform has been established for drug screening and testing [110].

PD is the most common movement disorder; it is characterized pathologically by the accumulation of Lewy Bodies consisting of insoluble aggregates of α-synuclein and reduced dopamine levels due to degeneration of the substantia nigra [91, 111]. Previous studies have identified a number of PD risk genes including *SNCA*, *PARK2*, *PINK2*, and *LRRK2* [112]. In 2D cell culture, inducing hiPSCs into neurons and astrocytes, the degeneration of PD neurons was found to be associated with the accumulation of toxic α-synuclein in astrocytes [113, 114]. In cultured midbrain organoids from PD patients with the LRRK2-G2019S mutation, high-content imaging data has shown decreased dopaminergic differentiation, altered mitochondrial morphology, and increased cell death compared to the organoids from isogenic lines [102]. PD organoids can also be established by CRISPR-Cas9-mediated gene editing in human embryonic stem cells. After introducing the DNAJC6 mutation in human midbrain-like organoids, PD pathologic features such as midbrain-type dopamine neuron degeneration, pathological α-synuclein aggregation, an increase in intrinsic neuronal firing frequency, and mitochondrial and lysosomal dysfunctions were detected [115].

ALS is a neurodegenerative disease specifically affecting motor neurons; it typically results in progressive muscle atrophy and usually death from respiratory failure [92, 116]. Cultured motor neurons from ALS patients show degeneration, abnormal protein aggregation, and increased cell death [117, 118]. Given the unfeasibility of obtaining presymptomatic samples, the development of brain organoids may help elucidate initial molecular events. By using a combination of single-cell RNA sequencing and biological assays, it has been revealed that the cortical organoids from patients with ALS overlapping with frontotemporal dementia harboring the *C9ORF72* hexanucleotide repeat expansion mutation exhibit distinct transcriptional, proteostasis, and DNA repair disturbances in astroglia and neurons [119]. Apart from the traditional method of culturing brain organoids, ALS can be modeled by sensorimotor organoids, which contain sensory neurons, astrocytes, microglia, and vasculature, and include functional neuromuscular junctions (NMJs), and this ALS organoid displays impaired NMJs [103].

HD is the most frequent autosomal-dominant neurodegenerative disease; it is caused by somatic expansion of CAG repeats in the *Huntington* (*HTT*) gene, which results in neurodegeneration in the striatum and cortex [120, 121]. Patients with HD show motor, cognitive, and mental abnormalities in mid-life [122]. Fetal brains of HD patients and mutant mouse models display mislocalization of mutant huntingtin and junctional complex proteins, defects in neural progenitor cell polarity and differentiation, abnormal ciliogenesis, and changes in mitosis and cell-cycle progression [123]. By comparing HD organoids with controls at the transcriptome level, HD organoids had a more immature transcription profile as well as disrupted cortical cytoarchitecture, indicating a possible connection between mutant huntingtin and abnormal neural development [124]. Another study found that heat shock transcription factor 1 (HSF1) accumulates in the mitochondria of HD cell models, a mouse model, and human striatal organoids derived from induced HD iPSCs, and suppressing the mitochondrial localization of HSF1 by interfering with its binding to dynamin-related protein 1 can rescue the pathological HD changes in striatal organoids [104]. Taken together, organoids have become a powerful model in which to explore the pathogenesis and develop potential treatments for NDDs.

To date, there is no satisfactory treatment for NDDs. Many drugs are effective in laboratory animals but do not achieve the desired therapeutic effect in humans, most likely due to inherent differences between humans and other species. Thus, human brain organoids can be used as a compensatory approach for target identification and drug development. Previous studies have built a complete experimental system for drug screening for cancer using organoid technology [125–127], but the application of organoids in NDDs is more challenging because of the complexity of the origin of neuronal cell types, cell-type specific pathogenesis, and complicated cell-cell interactions, as well as the involvement of environmental and immune factors. Nevertheless, brain organoids resemble some key features of NDDs and thus can be used to test the effects of potential modulators. For example, treatment with the LRRK2 inhibitor 2 has shown some rescue effects on LRRK2-G2019S-dependent dopaminergic phenotypes in PD organoids [102]. The BBB is another element that should be considered because most NDDs at late stages are accompanied by disruption of the BBB, which fails to prevent the entrance of toxic substances from the circulatory system into the brain [25]. However, the current brain organoid models lack BBB structure, which limits their applications.

## Psychiatric Disorders

Because the clinical manifestations of psychiatric disease are very subjective, most being diagnosed by doctors through oral communications with patients, and objectively quantifiable phenotypes [128]. Thus, simulating mental diseases using animal models is extremely challenging. Owing to the research on the family pedigrees of patients with psychiatric diseases and the development of gene technology, many risk genes for psychiatric diseases have been identified, and various experimental models based on these genes have been established [128].

Mental diseases mainly include depressive disorder, schizophrenia, and bipolar disorder (Fig. 2). Major depression is one of the most common mental diseases; patients often present with loss of interest or pleasure, insomnia or hypersomnia, and mental disorders [129, 130]. However, the current understanding of the mechanism of depressive disorder is still incomplete, and some symptoms and etiology have great heterogeneity. In postmortem brain scans of people with depressive disorder, it has been found that GABA receptor-mediated inhibition is dysregulated in depressed individuals with a history of suicidal behavior, but the molecular mechanism underlying this abnormality is not clear [131, 132]. Recent studies have used hiPSCs from patients with a depressive disorder to induce the ventral forebrain organoids and GABAergic neurons *in vitro* [133]. Through transcriptome sequencing and single-cell technology, it has been found that the decreased expression of serotonergic receptor 2C in neurons under the condition of depressive disorder may lead to defective neuronal activity, and targeting the 5-HT2C receptor by adding small molecule agonists and genetic methods can effectively restore neural activity [133].

Schizophrenia is a chronic brain disease that occurs mostly in early adulthood. It is polygenic and is hypothesized to be a neurodevelopmental disorder with an as-yet-unknown molecular origin [134–136]. Disordered neurogenesis, impaired synaptic transmission, and dysfunction of mitochondria have been identified in neurons derived from hiPSCs from patients with schizophrenia [137–141]. The astrocytes induced by schizophrenic hiPSCs show DEGs related to inflammation and synaptic function, and transplanting schizophrenic astrocytes to mouse brains results in behavioral changes in cognitive and olfactory functions [142]. The reduction in synaptic density in schizophrenic patients is caused by the excessive synaptic pruning by microglia [143]. In an *in vitro* model of microglia-mediated synapse engulfment, schizophrenic microglia increase synapse elimination [144]. Recent studies have used hiPSCs from patients with schizophrenia to construct whole-brain organoids [145, 146]. Using transcriptome sequencing analysis, the authors found that the genes related to mitochondrial function showed marked differences [145]. By applying the Seahorse Mito Stress test of organoids, it has been found that the level of oxygen consumption of schizophrenic organoids decreases significantly [145]. These organoids also show neuronal dysfunction as reflected by weakened responses to electrical stimulation and KCl depolarization measured with a microelectrode array [145]. By analyzing the single-cell RNA sequence profile of schizophrenic organoids, decreased progenitor survival and disrupted neurogenesis were detected [147]. Transcription factor BRN2 and growth factor PTN have been identified as mechanistic substrates of neurogenesis and cellular survival, respectively, in schizophrenic organoids [147].

Bipolar patients experience recurrent episodes of depression and mania that affect perception, emotion, thought, and social behavior [148]. Due to the complexity of the symptoms, the diagnosis and treatment of bipolar cognitive disorder are still subjective. In cultured neurons derived from bipolar hiPSCs, mitochondrial abnormalities, hyperexcitability, anomalous calcium signaling, impaired neural differentiation, and decreased proliferation have been reported [149–151]. By transcriptome analysis of brain organoids derived from the hiPSCs of bipolar patients, it has been found that the expression of genes related to cell adhesion, neurodevelopment, and synaptic regulation is decreased, while the expression of genes related to immune signaling is increased in bipolar organoids [152]. In another study, bipolar organoids showed specific deficits in response to stimulation and depolarization, as reflected by neuronal activity measured by microelectrode arrays, and enrichment of endoplasmic reticulum pathways as analyzed by Gene Ontology analysis for DEGs [152]. In conclusion, organoids provide an *in vitro* model in which to study psychiatric disorders and help explore the causes and develop treatment strategies.

## Brain Tumors

Brain tumors are a type of neoplasm that arises from brain tissue or systemic cancer metastases, classified as malignant and benign tumors [153, 154]. Malignant brain tumors cause high morbidity and mortality and thus are considered one of the most devastating neoplasms due to the complexity of cell types and structures in the human brain. In 2021, the World Health Organization published the fifth edition of the classification of brain tumors, which recapitulated their characteristics and linked the molecular mechanisms of a set of brain tumors including gliomas, glioneuronal tumors, and neuronal tumors [155].

For a better understanding of brain tumors and the associated therapies, genetically engineered mouse models (GEMMs) have been established and used for mimicking the pathological phenotypes and elucidating the innate mechanisms of brain tumors [156–158]. However, GEMMs cannot fully simulate the phenotypes because of the species differences. Patient-derived xenografts (PDXs) are a model that transplants dissected tumor tissue from cancer patients into immunodeficient mice to mimic tumor growth *in vivo* [159, 160], and have been used for anti-tumor drug screening [161]. Nevertheless, PDXs lack the procedures for tumor origination and formation, which limits their application. Recently, the production of organoids has enabled further advances in brain tumor research [162]. Brain tumor organoids are classified by the origin of tumor cells such as genetically-engineered stem cells or dissected tumor tissues or cancer stem cells. For example, Bian *et al.* [163] established a neoplastic cerebral organoid from hESCs using genome-editing technologies to introduce mutation of tumor-suppressor genes combined with Sleeping Beauty transposon gene insertion. Tumor overgrowth occurs in induced cerebral organoids and mimics brain tumor formation. The MYC gene, a proto-oncogene, is over-expressed in cerebral organoids; it is exhibited in brain tumors such as glioblastoma (GBM), central nervous system primitive neuroectodermal tumor, atypical teratoid/rhabdoid tumor, and medulloblastoma. Another human cerebral organoid modeling GBM has been developed by targeting an HRas^G12V^-IRES-tdTomato sequence into the TP53 locus using the CRISPR-Cas9 system [164], which exhibits an increased fraction of tumor cells, accompanied by the expression of the GBM stem cell markers OLIG2, GFAP, and SOX2, and the proliferation marker KI67, indicating the GBM identity. Transplantation of brain tumor organoids into immunodeficient mice shows the invasiveness of the brain tumor and higher mortality of mice [164].

To overcome the limitation of the lack of a “normal” human brain microenvironment, Linkous *et al*. [165] have established a cerebral organoid glioma (GLICO) model system in which they introduced patient-derived glioma stem cells (GSCs) into hESC-derived cerebral organoids. In the GLICO system, GSCs invade, proliferate, and form tumors within the host organoids, and these processes faithfully phenocopy patient GBMs. Furthermore, the sensitivity to chemotherapeutic agents and ionizing radiation of GLICO tumors also confirmed it as a suitable model resembling *in vivo* tumors compared with 2D cultured tumor cells. Considering the cellular and genetic heterogeneity in inter- and intra-GBM samples, a comprehensive study has established patient-derived glioblastoma organoids (GBOs) and transplanted them into immunodeficient mouse brains [166]. These GBOs have been used experimentally for personalized drug testing, in particular T cell immunotherapy. This offers a possible approach to the development of patient-specific treatment strategies. By combining single-cell transcriptomics and live imaging of primary tumor resections, multiple GSC subtypes have been identified, leading to the finding of an invasive population similar to outer radial glia (oRG), a type of cortical neural progenitor which is believed to contribute to cortical expansion and folding [167]. Transplanting GFP-labelled oRG-like tumor cells into hPSC-derived cortical organoids confirmed the tumorigenic and invasive properties of this tumor cell population [167]. Thus, the analysis of GSC heterogeneity may provide an optimized strategy for an individual patient.

Brain tumor organoids have been applied to studies of GBM, medulloblastoma (MB), and meningioma [168] (Fig. 2). MB is the most common malignant brain tumor in the cerebellum that occurs mostly in childhood [169]. Transcriptional profiling has revealed four main subgroups of MB, and group 3 is characterized by c-MYC upregulation and has the worst outcome [170]. Based on the previous protocol of cerebellar organoid generation [171], a group 3 MB organoid model has been developed by overexpression of the *Otx2* and *c-MYC* genes, which promote tumor cell over-proliferation [172]. Using the MB organoids, the authors found that up-regulation of the on-co-suppressor *SMARC4* or treatment with Tazemetostat, an EZH2-specific inhibitor, reduces Otx2/c-MYC-induced tumorigenesis [172]. In another study, overexpression of the MB driver genes *MYC* and *Gfi1* in human cerebellar organoids induces group 3 MB with an epigenetic profile similar to human patients *in vivo* [173]. Moreover, activation of the Notch1 pathway fosters group 3 MB formation [173]. Meningiomas are the most common intracranial tumors, but the molecular drivers are poorly understood and effective treatments are lacking because of the shortage of research models. Recently, several studies have established meningioma organoid models from patient-derived tumor cells or tissues [174–176]. The histological features and innate molecular profiles of meningioma organoids are similar to corresponding parental tumors, and this advantage has allowed the model to be applied to the identification of potential targets for meningioma therapy [174].

## Outlook

Improvements in organoid technology in recent years have contributed greatly to our understanding of the mechanisms of human brain development and the pathogenesis of neurological diseases. However, *in vitro* culture conditions limit the size of the organoid, neuronal maturation, and subsequent production of more complete cell types, such as astrocytes and oligodendrocytes. Furthermore, the lack of blood vessels and immune cells also limits the applications of brain organoids. Although some advances have been made, the innermost parts of an organoid eventually die due to the lack of oxygen and nutrients. Several studies have attempted to establish assembled organoids to resemble inter-regional interactions in the brain and brain periphery interactions, but they only partially mimic the counterparts of the real human body. Nevertheless, partial simulation has already demonstrated broad prospects in disease modeling and the clarification of mechanisms, as well as drug screening or testing. Transplantation of specific types of brain organoids into injured or degenerative regions provides another avenue for the repair of the related neural circuits under disease contexts. A combination of multidisciplinary strategies would help optimize the brain organoid system and broaden its applications.
